# Mr. Hill, on Medical Reform

**Published:** 1807-01

**Authors:** G. N. Hill

**Affiliations:** Chester


### *To the Editors of the Medical and Physical Journal.*

Gentlemen,

I AM one of those who have long entertained the opinion, that, before any individual, or any body of men, set about regulating the affairs of the world at large, it is incumbent on them to examine with strict impartiality those of their own house; and by the introduction of salutary well-timed reform, deprive any invidious sneerer of the illnatured natured satisfaction of observing, "behold the beam in thine own eye, and endeavour to remove it, before thou censurest thy brother for retaining his beloved mote."

The subject of medical reform is doubtless a very important one; I may say, without fear of contradiction, exceeded by none, inasmuch as the public health and welfare are so intimately connected with its just and necessary regulations; I humbly conceive, that all attempts to produce radical *external* reform should be erected upon the best possible foundation, adapted to the temper of the times, and the advancement and diffusion of knowledge, the reformers being themselves the first to set forth good examples, and well convinced they do not individually transgress against those very regulations they appear so zealously anxious to establish for the conduct of others; this foundation ought to be internal.

The conduct of medical officers in our public hospitals; their introduction to them; the laws of these establishments, as they relate to practitioners of medicine; the interchange of knowledge and communication of improvement between the several bodies of medical men attached to our cities, towns, and schools of medicine; the abandonment of long existing approaches to charlatanism, with many other matters, all require salutary reform; it is almost impossible to comprehend every article in a letter of this kind, which invites examination, or suggests the want of gradual improvement, the hydra has too many heads to be cut off at once; but when a good beginning is once fairly made, it is at least within probability, that a wholesome termination, adapted to present times, may be obtained.

Upon a very cursory inquiry, it will be found, that one public institution holds a law most sacred, which was enacted half a century past, enjoining that no individual shall presume to attempt to practice the art of surgery upon the objects which this establishment thinks proper to select as its elemosynary dependants, unless he religiously abstain from pharmaceutical practices; or, at least, that he suffer not the governors of the endowment to be witnesses, that he combines with this noble art the indispensible, and absolutely inseparable knowledge of pharmacy and its practice; and that he dares to do his employers the justice of actually dispensing, or at least superintending, the dispensation of those medicines; on the goodnes of which, and their accuracy of preparation, their lives so often depend. But if this said officer of health, subsequent to induction, do do actually, though not avowedly, practice as physician, surgeon, and apothecary, by attending every medical case to which he may be called, writing a prescription, and committing it to the care of any idle, ignorant, or dissipated novice in the nearest druggist's shop to compound; he commits no infringement on the wise regulation contained in the laws of the house, and is at full liberty thus to acquire a very principal, generally a major part, of his future fortune, provided he be at all times scrupulously careful not to defile his fingers with rolling pills, compounding mixtures, or rubbing electuaries; thus he daily receives the fee of the physician, and part of the profit of the apothecary, and what is often of far more consequence than hospital practice, he gets better and more certainly paid than either of them. In consequence of laws similar to these being still suffered to disgrace the statute books of some benevolent institutions, the public daily witness young men just emerged from the anatomical school and lecture-room elected as surgeons to them, merely because they solemnly assure the donors that they will not practice pharmacy; yet scarcely one has left the election room twenty-four hours, who, if called to a small-pox patient, would or does refuse to attend the full event of the case. Here then is room for reform. On this subject it might be asked, ought not every mite of individual talent to be brought to the altar of Hygeïä? Which are the best and most natural channels for the public to receive the benefits derivable from these offerings but the public hospitals, dispensaries, &c. where all diffused knowledge ought to centre, as in a common emporium, and be thence distributed to bless mankind; instead of which, those members of our profession, who profess to do least, in the least generally useful manner, and make a display of their confined circle of qualified ability, and who at the same time are, from early age and want of experience, the least qualified, are those preferred.

Another institution declares, by *modern* law, That no surgeon so called, shall presume to offer himself as a candidate for hospital honours, unless he can produce a diploma from the Royal Corporation of Surgeons in London. Whatever may be his merit, (for that in truth is not always the question) this must be his passport to the road of doing good, this is the "note of preparation" to his becoming the servant of the poor and helpless of the community. Now, gentlemen, I need not tell persons of your general information, that in a class of one hundred young men attending attending the justly celebrated lectures of a Cline or a Cooper, that half of them are seldom rich in any thing but industry, attention, and ability; probably, with all their care, and the unremitted exercise of every prudential rule, can with difficulty complete two written courses of lectures with honour, and pay their necessary debts. Many of these possess undoubted medical ability, and yet are not blessed with a sufficient sum to obtain the college credentials the generous supporters of the hospital require; so that, alas! that poverty so honourable, and which is but too often the companion of merit, is thus destined to exclude them from sharing the honours and satisfaction attending the public service of a populous institution, where their knowledge, and habits of close attention, might prove of the utmost consequence to its best interests. Where merit actually exists, it certainly ought to claim (independant of any other qualification, and unconnected with any unmanly condescensions) an unembarrassed title to enter the road of public fame for preferment; hence the certificates of celebrated teachers should be a sufficient diploma for the poor, but often worthy scholar, these being the most fairly and honourably attained; while the diploma of a college is often granted more through chance, than from a clear determined knowledge of the ability of the candidate. Public examinations must necessarily be short; the examined and the examiners utter strangers to each other, and the questions put by the latter, the fruit of accident. The answers of the former may perchance be correct and fortunate, where sterling ability is wanting; but being correct he obtains his diploma; whereas diffidence, with the possession of extensive science, has been dismissed, from the surprize of some uncommon question being put, in an unlucky moment of confidence, being overcome by a deficiency in necessary assurance.

We are erring mortals, and are taught to pray daily against being led into temptation; there should be no toll-gate to college, or diplomatic honors, no pecuniary emoluments attached in any shape to the office of judges of medical merit, nor to the attendance of practitioners in hospitals; a testimonial of regular pupillage, learned ability, moral conduct, and the certificates of lecturers, ought to entitle all to a diploma, without more expence than a small gratuity to the secretary or clerk, for filling up and registering the credential. Here there is room for reform.

There has long existed an absurd attempt to keep up an unmeaning distinction between the practice of surgery and medicine, so far as it concerns surgeons and apothecaries. It is high time, for the honour of the profession, and the good of mankind. that it should be done away ; the folly of the attempt daily made to draw a clear line between these practitioners, and those who affect to stile themselves by the epithet regular Surgeons, is apparent to the philosophic and reflecting part of the world ; nature herself has otherwise ordained this matter ; whoever saw a purely surgical case ? Do not the subjects of the highest health, on receiving an unfortunate accident, requiring the aid of operative surgery, require that of medical surgery also ? And with respect to all chronic cases, the combination of talents is still more necessary ; it were waste of time to adduce instances, but it may be worth while to enquire, where is the surgeon-apothecary (especially in the country) of long standing, who has not prolonged many a life, and saved many a limb, from this combination of talents ? A veteran of the class of regulars, as they are called, chose, in a ludicrous vein, to observe, " that the man who handled a mortar was not competetent to perform a surgical operation." And yet this veteran practitioner is truly a surgeon-apothecary, if attending every purely medical case that offers, constitutes that character ; for it is superfluous to observe, that there are pure medical, though not surgical cases. Now these almost non-descripts cannot be called physicians, merely because they imitate prescriptions of the composition of a powerful medicine, formed of the best materials. But the veteran could certainly only mean, that they who had just been employed in beating a mass of lead pills, or rubbing the refractory ingredients of an electuary, was not fit to be immediately transformed to the wielding of the lancet, or the direction of the couching needle, from the temporary tremulus under which it must labour, after being employed in these servile, but necessary occupations. Granted, so far as it concerns those who are their own menial servants, which I presume is a matter of very rare occurrence. As to the education of the whole mass of pupils who annually migrate to the metropolis, to Edinburgh, &c. it is nearly the same, physicians excepted ; and doubtless the man who says, " O, I confined myself wholly to Anatomy and Surgery, I know nothing of medicine, I leave that to the regular Physician," may be stiled a Surgeon ; a regular surgeon may be diplomated over and over again, but he never can be a thooughly useful servant of the public, until he be reduced to the the office of a mere mechanical labourer, as in days of other times, until Surgeons again become mere gross operators on the human frame, directed by the Physician, as the bellows-blower is by the organist. What has emancipated the exalted art from this servile obedience but a knowledge of medicine? And who are the most successful operators, but those who understand best the resources and just application of the powers of medicine? Are not the knife, which recovers the shattered limb, and the bark which arrests the latent mortification, joint-instruments in the hands of the qualified artist? The operations in Surgery are merely mechanical, and too frequently exist as opprobria, rather than redounding to the honour of the art; no man of common sense, for an instant, draws a comparison between that ability which prevents the necessity of his leg being amputated, and he who deprives him of his limb with superior exactness and unusual dispatch. All the resources of acquired information must be brought into exercise in the former case; in the latter, correct anatomical knowledge is the almost only requisite; but what modern surgeon contents himself with this mere mechanical information? or what honest man is satisfied with the most dextrous hand, and keen eye, in performing the operation for femoral or scrotal hernia, independant of a knowledge of the nature and treatment of peritoneal inflammation? Yet it cannot be denied, but a mere anatomist might perform these operations very safely, and thus become a strictly regular Surgeon. Ignorant of the power of medicine, when duly co-operating with the latent resources of nature, joined to a silly desire of appearing dextrous operators, have stimulated many a rash young man to commit serious crimes against the happiness, existence, and usefulness of his fellow-creatures. Here there is room for reform.

It has long been greatly repugnant to my feeling, that any man should ever run the immense risque of prescribing any medicine at random, more especially since we have become so fond of those which are designated as poisons. That this is daily done, is verified by many known melancholy proofs, to say nothing of the concealed results of blunders and accidents. But, Gentlemen, to spread a passion for reform is at any time easy; the love of novelty and of change, incident to human nature, is readily excited to action, but of extreme difficult direction in obtaining a salutary result; nor is it easy to keep the spirit of reform within the bounds of justice, utility, independence, and truth, assisted with self-interest, and leavened with private dislike, it is too prone to defeat its own intentions. How far the plan now in agitation is calculated to promote the best interests of the public, and the honour of the art, I presume not to say; but it appears to my mind much too external, too superficial; and that before application be made to the legislature, and useful alterations be submitted to general consideration, *e. g.* ought it not to be considered? If the Physician be by government authorized legally to recover his fee, why are the profits of the Surgeon-apothecary, the bulk of whose business is in prescribing and directing medicine, to depend upon the quality of the articles he sends to his patient? And why, after a lapse of years, is he compelled, when payment is refused by a bad man, to prove the delivery of every item of an account, consisting of an hundred articles, no one of which reaches to three shillings in account? Such evils as these are disadvantageous, and most degradingly dishonourable to the practitioners, of what ought to be the most liberal and useful of the professions.

I am, &c.

